# Assessment of the Sex Hormone Profile and Its Predictive Role in Consciousness Recovery Following Severe Traumatic Brain Injury

**DOI:** 10.3390/life15030359

**Published:** 2025-02-25

**Authors:** Seyed Ahmad Naseri Alavi, Sajjad Pourasghary, Amir Rezakhah, Mohammad Amin Habibi, Aydin Kazempour, Ata Mahdkhah, Andrew Kobets

**Affiliations:** 1 Department of Neurosurgery, School of Medicine, Emory University, Atlanta, GA 30332, USA; 2 Faculty of Medicine, Urmia University of Medical Sciences, Urmia 5714783734, Irandrrezakhaha@gmail.com (A.R.); akazempour@gmail.com (A.K.); amahdkhah@gmail.com (A.M.); 3 Department of Neurosurgery, Shariati Hospital, Tehran University of Medical Sciences, Tehran 1474833163, Iran; mohammad.habibi1392@yahoo.com; 4 Department of Neurological Surgery, Montefiore Medical, Bronx, NY 10467, USA; akobets@montefiore.org

**Keywords:** severe brain trauma, consciousness disorder, sex hormones, gender difference, recovery, brain injury

## Abstract

Introduction: Traumatic brain injuries (TBIs) are conditions affecting brain function caused by blunt or penetrating forces to the head. Symptoms may include confusion, impaired consciousness, coma, seizures, and focal or sensory neurological motor injuries. Objective: This study evaluated sex hormone profiles and their predictive role in returning consciousness after severe traumatic brain injury. Materials and Methods: We included 120 patients with TBIs and collected comprehensive information about each patient, including the cause of the trauma, age, gender, Glasgow Coma Scale (GCS) score, Injury Severity Score (ISS), and neuroradiological imaging data. The ISS was used to assess the severity of the trauma. At the same time, the lowest GCS score was recorded either before sedation and intubation in the emergency room or by emergency medical services personnel. For female participants, samples were collected during the luteal phase of the menstrual cycle (days 18 to 23). Results: The mean age of male patients was 33.40 years, ranging from 23 to 45 years, while female patients had an average age of 34.25 years, ranging from 25 to 48 years. The primary cause of injury for both genders was motor vehicle accidents. In male patients, testosterone levels were significantly higher in those classified as responsive (RC) compared to those non-responsive (NRC), with levels of 2.56 ± 0.47 ng/mL versus 0.81 ± 0.41 ng/mL (*p* = 0.003). A cut-off point of 1.885 ng/mL for testosterone levels in males was established, achieving a sensitivity and specificity of 86.7% and 86.7%, respectively. In female patients, progesterone levels were elevated in those who regained consciousness, measuring 1.80 ± 0.31 ng/mL compared to 0.62 ± 0.31 ng/mL (*p* = 0.012). A cut-off point of 1.335 ng/mL for progesterone levels in females was determined, with a sensitivity and specificity of 93.3% and 86.7%, respectively. Conclusions: We can conclude that sex hormone levels in the acute phase of TBIs can vary between males and females. Notably, serum testosterone levels in males and progesterone levels in females with TBIs are significant prognostic factors for assessing the likelihood of regaining consciousness after such injuries. These findings underscore the importance of considering sex hormone profiles in TBI recovery prognosis.

## 1. Introduction

Traumatic brain injuries (TBIs) are a condition characterized by dysfunction of the brain resulting from either blunt or penetrating forces applied to the head. This injury may manifest as confusion, altered levels of consciousness, coma, seizures, and specific sensory or motor neurological impairments [1]. TBIs commonly occur following traffic accidents, falls from heights, sports injuries, and physical violence, which are almost always among the most common presentations to emergency departments. TBIs are one of the most common causes of morbidity and mortality in people under 45 years of age worldwide [2]. The leading causes of TBIs in the United States are falling from heights (32%), motor vehicle accidents (19%), injuries from accidents and natural disasters (18%), and street fights (10%) [3]. TBIs mainly present as primary (mechanical causes) or secondary (late non-mechanical causes) brain injury, involving local or global changes in metabolism and blood flow, neurotoxicity, cerebral edema, neuronal inflammatory processes, and neuropsychological impairment [2]. Patients with acute severe TBIs often develop impairments, including reversible impairment of consciousness within 1 month of injury [4]. Early recognition of impairment of consciousness in patients with TBI can predict recovery of neurological function; faster recovery of consciousness is almost directly related to good long-term neurological outcomes [5].

The estimated prevalence of TBIs is 538, 235, and 322 cases per 100,000 population in the United States, Europe, and Australia, respectively [3,4,5]. In the United States, the highest prevalence of TBIs occurs among children aged 0 to 4 years, with a frequency of 1188 cases per 100,000. However, the highest rates of hospitalization and mortality due to TBIs are observed in the elderly and adults over the age of 65, with frequencies of 234 and 38 cases per 100,000, respectively [6]. In 2018, the prevalence of TBIs in Iran was reported to be 100 cases per 100,000 people. A study conducted in Urmia, Iran, in 2013 found that the highest prevalence of TBIs occurred in the 20–29 age group [7,8]. The prevalence of TBIs is higher in males than females, with a prevalence rate of 2 to 2.8 times more significant and a mortality rate that is 3.5 times higher [9].

Numerous studies have shown varying results regarding how gender affects the recovery and prognosis of patients. In investigations involving individuals with mild TBIs, females typically demonstrated less favorable outcomes in most study groups. Furthermore, additional research has suggested that both being female and having reached menopause act as both risk and protective elements [10,11,12,13,14]. Most post-traumatic complications in patients with TBIs tend to affect females. However, most clinical and laboratory studies on animals have focused primarily on males. Additionally, experimental evaluations using TBIs animal models have shown that female gonadal hormones may have protective effects [15].

Recent evidence indicates that estradiol significantly enhances excitotoxicity, particularly concerning glutamate, facilitating neuronal lactate degradation, promoting cerebral blood flow, and reducing apoptosis within the central nervous system (CNS) [16]. Over the past few decades, progesterone and its primary metabolite, allopregnanolone, have been recognized for their important and potent effects in alleviating cerebral edema. Additionally, research has shown that progesterone can mitigate neuronal inflammation, enhance blood–brain barrier integrity, and decrease oxidative damage. Recent clinical investigations have also confirmed that administering therapeutic doses of progesterone is safe and may benefit patients experiencing moderate to severe TBIs [17].

Hormonal imbalances, known as post-TBI hormone deficiency syndrome, are a prevalent issue during the acute stage of TBIs. Recent research has shown that over 80% of TBI patients experience some level of acute pituitary insufficiency and associated hypogonadism. These hormonal imbalances play a crucial role in the post-TBIs recovery process [18,19]. The study of how gender differences affect the nervous system’’s reaction to traumatic injuries is an evolving area. Current laboratory investigations continually demonstrate that female brain tissue experiences less damage in cases of TBIs when compared to males, a result believed to be associated with the effects of gonadal steroid hormones present at the time of the injury. However, the effects of gender on the outcome and recovery of TBIs remain uncertain. The current research sought to assess the sex hormone profile and its potential role in predicting the restoration of consciousness following severe TBIs to improve patient care and outcomes.

## 2. Material and Methods

### 2.1. Study Populations and Definitions of Variables

We enrolled TBI patients referred to the emergency department (ED) of Imam-Khomeini Hospital, Urmia, Iran, from January 2020 to January 2022. Using G*Power v3.1, considering an alpha error of 5% and a power of 90%, 60 patients were selected as the case group (return of consciousness [RC]) and 60 patients as the control group (without return of consciousness [NRC]), with 30 males and 30 females in each group. Therefore, the final sample size was 120 patients. The inclusion criteria were TBI patients aged between 18 and 75 who experienced head trauma, with a GCS score of 3–8 upon arriving at the ED. Trauma severity was stratified using both GCS scores (severe TBI: GCS 3–8) and ISS scores (major trauma: ISS > 15) [20]. Only front-seat passengers were eligible, and participants must not have been drivers. Additionally, those included in the study should have been unable to open their eyes for 24 h after admission. Participants were required to have no prior history of neurological disorders, breast cancer that needed chemotherapy or tamoxifen treatment, or any malignancies related to the pituitary, hypothalamus, or prostate that involved orchidectomy treatment. Moreover, candidates should not have any untreated thyroid conditions, and female participants in the follicular phase of their menstrual cycle were excluded from the study. Women were assessed for their menopausal status and childbirth history. In cases where this information was unavailable, individuals over the age of 50 were considered postmenopausal. The Ethics Committee of Urmia University of Medical Sciences has approved the present study, code (IR.UMSU.REC.1398.462).

### 2.2. Patient Characteristics and Hormonal Evaluations

Patient information, including the cause of trauma, age, gender, ISS, GCS scores, and neuroradiological imaging data at baseline, was recorded on a standardized case collection form. The ISS score was used to assess the severity of the trauma. The ISS was calculated using the Abbreviated Injury Scale (AIS). Each body region (e.g., head and thorax) was assigned an AIS score (1–6), and the ISS was derived as the sum of the squares of the three most severely injured regions [21].

The lowest GCS score recorded in the ED before sedation and intubation or documented by EMS personnel was considered. A head computed tomography (CT) scan was utilized to determine the type of injury. Head injury type (e.g., subdural hematoma or diffuse axonal injury) was classified using non-contrast head CT scans and categorized according to the Rotterdam CT scoring system. Specific CT findings (e.g., midline shift or basal cistern compression) were recorded and scored as part of the Rotterdam classification [22].

Sex hormone levels in all patients were measured one week following the TBIs. For female patients, measurements were taken during the luteal phase of their menstrual cycle (days 18 to 23). Patients not in this specified menstrual phase were excluded from the study. Blood samples were collected from patients around 7:00 a.m. to measure estradiol, follicular stimulating hormone (FSH), luteinizing hormone (LH), progesterone, prolactin, and testosterone levels. Estradiol (Roche & Cobas Co., Mannheim, Germany; LOT#47073802), progesterone (Calbiotech Inc., El Cajon, CA, USA; LOT#OHP6340), and testosterone (Calbiotech Inc., USA; LOT#TES6616) levels were analyzed using the ELISA (enzyme-linked immunosorbent assay) (ELISA microplate reader, Biobase Co., Jinan, China), and FSH (DiaZist Co., Tehran, Iran; LOT#DFS907), LH (DiaZist Co., Iran; LOT#DLH910), and prolactin (Pishgaman Sanjech Co., Tehran, Iran; LOT#01-0501) levels were measured through the electrochemiluminescence immunoassay method (ARCHITECT i1000SR, Abbot Co., Baar, Switzerland) at the accredited clinical chemistry laboratory of Imam-Khomeini Hospital, Urmia University of Medical Sciences.

### 2.3. Outcome Definition

The primary outcome measured was the patients’ regaining of consciousness. Therefore, patients were divided into two categories: those who recovered consciousness (RC) and those who did not recover consciousness (NRC). The determination of RC patients was based on three criteria: (1) motor abilities, (2) social interaction capabilities, and (3) cognitive function in social contexts. The level of consciousness during periods of impaired awareness was evaluated using the Coma Recovery Scale-Revised (CRS-R) [23], and patients were monitored over 6 months.

### 2.4. Statistical Analysis

The Kolmogorov–Smirnov test first indicated the data’s normality. Descriptive statistics were employed to analyze the demographic details of the patients, presenting them as mean (SD) for normal distribution or as median with the 25th, 50th, and 75th percentiles for non-normal distribution. Quantitative variables were compared using an independent *t*-test for normal distribution or the Mann–Whitney U test for non-normal distribution. The comparison of qualitative variables was carried out using the chi-square test or Fisher’s exact test. We performed the logistic regression analysis to identify variables that predicted the return of consciousness. The regression model incorporated sex hormones and clinical predictors such as age, GCS and ISS scores, and head CT scan reports classified according to the Rotterdam CT classification. The patients’ return to consciousness prediction was executed by employing the receiver operating characteristic (ROC) curve method. A *p*-value < 0.05 was considered statistically significant. All two-sided analyses were conducted using SPSS software version 21.0 (SPSS Inc., Chicago, IL, USA).

## 3. Results

### 3.1. Patients Characteristics

We enrolled a total of 120 patients with severe traumatic brain injury from an initial pool of 4105 individuals referred to the emergency department. The average age of male patients was 33.40 years (ranging from 23 to 45), while female patients had an average age of 34.25 years (ranging from 25 to 48). The predominant cause of injury for both genders was motor vehicle (car) accidents. Upon hospital admission, the median GCS score for patients of both sexes was 5 (with a range of 3 to 6). The mean ISS was 37 (ranging from 30 to 42) for male patients and 36 (ranging from 29 to 42) for female patients. There were no statistically significant differences between the sexes concerning age, mechanism of injury, CT scan findings, GCS scores at the time of admission, or ISS scores. The baseline characteristics of the patients are shown in Table 1.

### 3.2. Comparison of Sex Hormone Serum Levels Between Males and Females

Table 2 summarizes the serum sex hormones single-factor analysis for male and female patients by RC and NRC groups. Here, we compared sex hormone serum levels between males and females in RC and NRC groups separately.

### 3.3. Comparison of Sex Hormone Serum Levels Within Each Gender Between RC and NRC Groups

Sex hormones (e.g., testosterone and estradiol) were compared within the same gender (e.g., males in RC vs. males in NRC; females in RC vs. females in NRC) to account for baseline physiological differences between males and females.

In patients both with and without regaining consciousness, testosterone levels were significantly higher in males, suggesting a potential role of testosterone in consciousness recovery. In males, testosterone levels were significantly elevated in the RC group compared to the NRC group, measuring 2.56 ± 0.47 ng/mL versus 0.81 ± 0.41 ng/mL (*p* = 0.003). However, in females, no statistically significant difference was observed in testosterone levels between RC and NRC, with values of 0.17 ± 0.08 ng/mL and 0.20 ± 0.08 ng/mL, respectively (*p* = 0.209).

Estradiol levels were notably higher in females who regained consciousness, indicating a potential role of estradiol in this process. However, there was no significant difference in estradiol levels between the sexes for patients who did not regain consciousness. In males, estradiol levels showed no significant difference between RC and NRC patients (20.16 ± 7.41 ng/mL versus 34.21 ± 7.74 ng/mL; *p* = 0.078). In contrast, estradiol levels in females were significantly lower in RC patients compared to NRC patients (80.96 ± 12.84 ng/mL versus 39.00 ± 13.70 ng/mL; *p* = 0.002).

FSH levels were significantly higher in females for patients with and without regaining consciousness, suggesting its potential role in consciousness recovery. In males, FSH levels were significantly higher in patients who regained consciousness (4.61 ± 1.37 mIU/L versus 1.93 ± 0.90 mIU/L; *p* = 0.001). Conversely, in females, FSH levels were significantly lower in those who regained consciousness (12.66 ± 5.00 mIU/L versus 23.53 ± 2.71 mIU/L; *p* = 0.002).

LH levels were significantly higher in females, both in patients with and without regaining consciousness, highlighting a possible role for LH in recovery. There was no difference in LH levels in males between those who regained consciousness and those who did not (3.27 ± 1.18 mIU/L versus 4.73 ± 0.96 mIU/L; *p* = 0.217). However, in females, LH levels were significantly lower in patients who regained consciousness (6.88 ± 1.19 mIU/L versus 9.98 ± 1.62 mIU/L; *p* = 0.003).

Progesterone levels were also significantly higher in females regardless of consciousness status, indicating a potential role in recovery. In males, progesterone levels were significantly lower in patients who regained consciousness (0.15 ± 0.05 ng/mL versus 0.33 ± 0.06 ng/mL; *p* = 0.029), while in females, progesterone levels were higher in those who regained consciousness (1.80 ± 0.31 ng/mL versus 0.62 ± 0.31 ng/mL; *p* = 0.012). Lastly, prolactin levels were significantly higher in females regardless of consciousness status, suggesting a potential role in recovery. In males, prolactin levels were significantly lower in patients who regained consciousness (22.09 ± 6.13 ng/mL versus 33.94 ± 4.12 ng/mL; *p* = 0.003), whereas in females, prolactin levels were higher in those who regained consciousness (60.90 ± 8.14 ng/mL versus 49.53 ± 12.10 ng/mL; *p* = 0.002).

### 3.4. Predictors of Recovery After TBIs

To analyze the role of hormones in predicting a patient’s state of consciousness, we employed logistic regression analysis, using the return or non-return of consciousness as the dependent variable. The results in Table 3 indicate that testosterone levels in males (OR = 4.798) and progesterone levels in females (OR = 7.930) significantly contribute to estimating the likelihood of consciousness returning in patients with TBIs.

**Figure 1:**
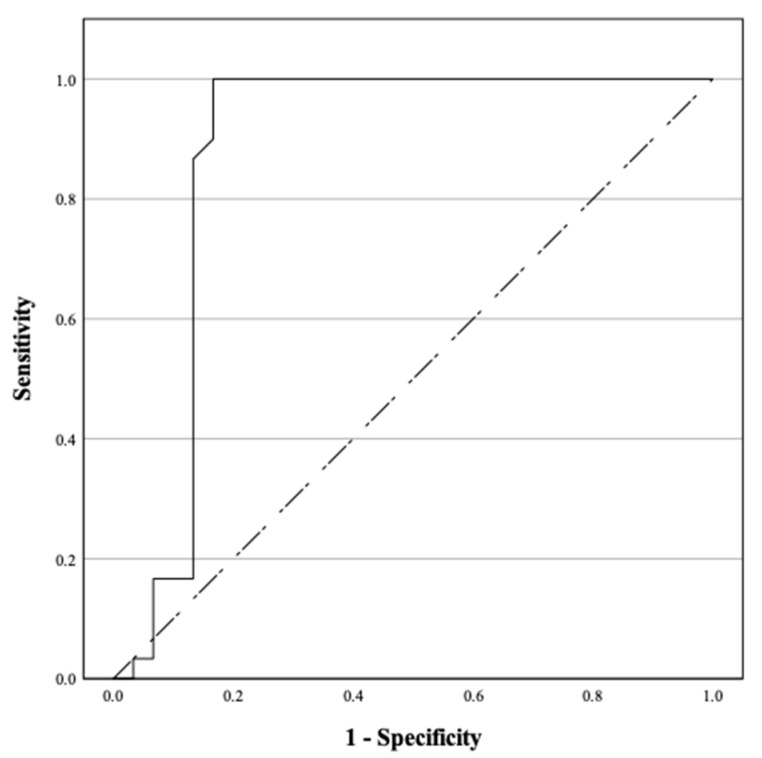
ROC curve for determining the diagnostic value of testosterone levels in males in assessing the state of consciousness of TBI patients.

**Figure 2:**
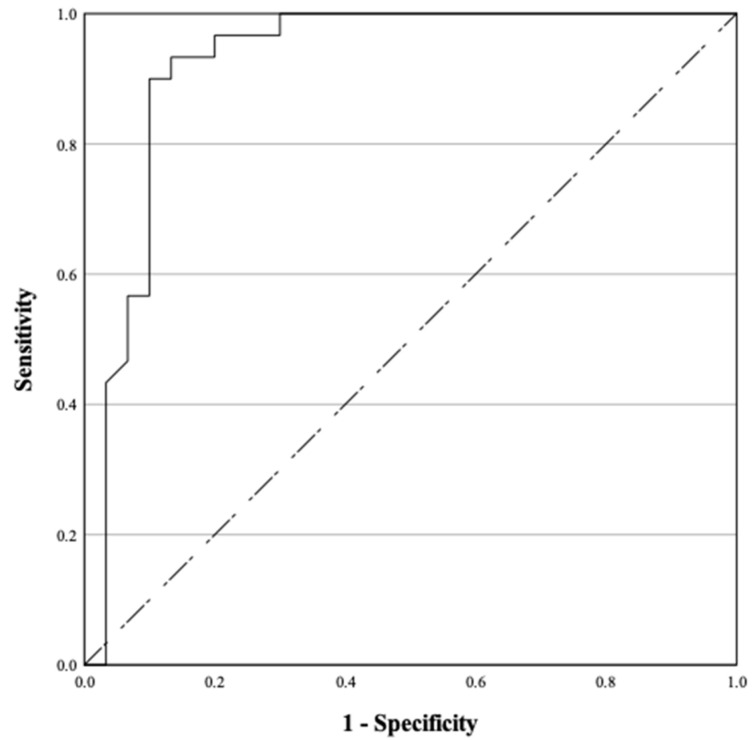
ROC curve for determining the diagnostic value of progesterone levels in females in assessing the state of consciousness of TBI patients.

The results were analyzed using Receiver Operating Characteristic (ROC) Analysis to evaluate the estimated values of testosterone and progesterone. The findings are illustrated in Figure 1 and Figure 2, respectively. From the analysis of Figure 1, the area under the curve (AUC) for testosterone was 0.875 (95%CI: 0.764–0.986). The optimal cutoff point for testosterone levels in males was 1.885 ng/mL, with a sensitivity of 86.7% and a specificity of 86.7%. In Figure 2, the AUC for progesterone was found to be 0.923 (95%CI: 0.844–1.000). The recommended cutoff point for progesterone levels in females was 1.335 ng/mL, demonstrating a sensitivity of 93.3% and a specificity of 86.7%.

## 4. Discussion

The alterations in pituitary hormone levels following TBIs have been reported in multiple studies. However, many of these studies failed to distinguish between male and female subjects. In our investigation, we focused on the variations in sex hormones and their implications based on the patients’ sex after TBIs [18,19]. Our results showed gender-specific differences in sex hormone concentrations during the acute phase following the injury. A key finding of this research was identifying the predictive significance of fluctuations in testosterone levels in males and progesterone levels in females regarding the recovery of consciousness for each gender.

According to recent studies, pituitary hypofunction is frequently seen in individuals following TBIs. However, the exact cause of this condition is not yet fully understood. The most widely accepted theory pertains to pituitary ischemia. Elevated intracranial pressure and swelling in the hypothalamic–pituitary region can also disrupt sex hormone levels following TBIs [24]. Consequently, surgical interventions during the acute phase of TBIs, such as decompression surgery, may alleviate hormonal imbalances by lowering intracranial pressure. There are also data suggesting that pituitary hypofunction in patients with TBIs might be missed due to the absence of routine hormone assessments [25]. Furthermore, most clinical research regarding pituitary function in TBI cases has predominantly involved males, given the higher occurrences of TBI in this gender compared to females [9,26]. According to this consequence, we enrolled equal sampling from both genders.

Our findings indicated significant differences among patients who regained consciousness after TBIs between the sexes in levels of testosterone, progesterone, estradiol, FSH, LH, and prolactin. Additionally, in patients who did not recover consciousness, significant differences in the same hormonal levels were noted between the sexes. The variations in estradiol, progesterone, and prolactin levels between the sexes regarding regaining consciousness were distinct. Therefore, these findings suggest that TBI has different impacts on hormone changes in men and women, and based on available data, the outcomes following TBI are not uniform across genders [10,27,28,29,30,31,32].

Experimental studies have indicated that female rats exhibit lower susceptibility to developing cerebral edema following TBIs. In contrast, male rats experience more severe cerebral edema in the same situations, potentially resulting in extensive brain damage. Clinical research has also revealed a greater survival rate for females after trauma and its related complications compared to males [10,27,28,29,33].

Conversely, some researchers have reported contradictory results, indicating worse outcomes and higher mortality rates for female patients [10]. Studies have also shown that the extent of brain tissue damage after TBIs tends to be less in females than in males [34]. The findings from various studies affirm the pathophysiological effects of these gender differences. Both clinical and laboratory investigations demonstrate the influence of sex hormones on brain injury. Thus, gender-related fluctuations associated with steroid hormones may impact the physiology and pathophysiology of TBIs [29,30,31,32].

The loss of consciousness following a TBI raises significant concerns for medical professionals and causes anxiety for the families of affected patients; nevertheless, the underlying mechanisms remain not fully elucidated, and patient prognosis under these circumstances is often unclear [27]. Consciousness is regarded as the primary function of the human cerebral cortex [35], and the ascending reticular activating system (ARAS) is crucial in managing consciousness levels [34]. It is believed that any dysfunction or injury to the ARAS components after TBIs—including areas such as the brainstem, thalamus, and cerebral cortex or disruptions in the white matter connections between the thalamus and the cortex—can result in decreased consciousness [36]. Furthermore, the hypothalamus plays a vital role in self-awareness and regulates sleep and awakening cycles, serving as the central controller of consciousness timing [37,38]. Dysfunction of the hypothalamic–pituitary axis due to TBIs typically results from damage to the hypothalamus, which can occur due to hypoxia, direct mechanical trauma, or vascular injury [19,25]. Consequently, based on the aforementioned research and our study findings, changes in hormonal levels after TBI may serve as indicators for predicting the restoration of consciousness in patients.

In our study, sex hormone levels were investigated as a predictor of the outcome of loss of consciousness in patients. The results showed that testosterone levels in males and progesterone levels in females were good predictors of return of consciousness. Our study also showed that normal or increased testosterone levels in males and progesterone in females were significantly associated with a reduced risk of loss of consciousness.

While the exact mechanism by which testosterone enhances consciousness remains unclear, various studies bolster our findings. Recent research suggests a link between lower testosterone levels and the severity of TBIs. In males, there is a direct correlation between plasma testosterone levels and GCS scores [39,40]. Furthermore, testosterone levels have been associated with both mortality and morbidity in patients with TBIs [41]. Clinical research suggests that male patients with TBIs may benefit from restoring serum testosterone levels [42,43]. Laboratory studies have also demonstrated the positive effects of testosterone following brain injuries. A study by Lopez-Rodriguez et al. found that brain testosterone levels were inversely related to TBI severity and swelling while showing a direct relationship with GCS scores. This research indicated that animal models exhibiting lower brain testosterone levels suffered from more pronounced neurological impairments [44]. Moreover, brain testosterone has been recognized for its neuroprotective properties against oxidative damage in experimental models [45]. Additional studies have shown that endogenous androgens may influence the ability of neural progenitor cells to produce neural precursors during oxidative stress [46]. Evidence suggests that steroid hormones could regulate neurogenesis in the adult subventricular zone by impacting the synthesis of brain-derived neurotrophic factors [47]. Additionally, it has been noted that testosterone might enhance memory performance in older rats by promoting the transfer of nerve growth factors from the hippocampus to the cortex [48]. We can indicate that testosterone levels may be a significant predictor of recovery.

Substantial gender differences are evident in the functional, morphological, and neurochemical outcomes of the brain despite the similarities between the sexes [49]. Research has highlighted sex-specific responses to traumatic injuries of the nervous system, providing evidence that links sex hormones with alertness in both males and females following TBIs [9,10,27,28].

To assess whether serum testosterone and progesterone levels effectively predict RC or NRC status in patients after severe brain injury, we investigated the diagnostic value of these hormones, and the results showed acceptable AUC values in ROC analysis. We also found that higher serum testosterone levels in males and serum progesterone levels in females following TBIs improve the possibility of consciousness recovery. In line with our findings, it has been reported that restoring serum testosterone levels to typical values in male patients can improve outcomes after TBIs [42]. Evidence suggests that traumatic axonal damage in the brainstem reticular activating system and thalamus and severe damage to the cerebral cortex cause loss of consciousness following TBIs [50]. In both sexes, androgens are an important factor in axonal regeneration [51]. A possible mechanism by which testosterone may improve the recovery of consciousness after TBIs is related to its function in facilitating axonal regeneration [52].

In contrast, systemic administration of testosterone to female animals has shown less axonal recovery, possibly due to the conversion of testosterone to estradiol by aromatase and the inability of estradiol to bind to androgen receptors in neurons [52]. On the other hand, the effect of sex hormones on the brain and behavior can be influenced by other factors, such as menopausal status, age, and parity. In the present study, samples were also taken from patients in the luteal phase of menstruation, and it seems that the relationship between the state of consciousness and sex hormones in females in previous studies is due to this; however, in the present study, progesterone levels were significantly associated with the recovery of consciousness in females. Recent studies have shown that progesterone can suppress inflammation in the nervous system; reduce edema, oxidative damage, and blood–brain barrier damage; improve dendritic arborization and synaptic regeneration; and limit cell necrosis after traumatic brain injury [18,53,54]. Although in vitro studies have shown the role of progesterone and estrogen in improving the recovery of TBI, clinical studies have not shown evidence of a beneficial effect of progesterone in patients with severe TBI. Also, there is no recommendation for the use of progesterone as an agent to improve the state of consciousness after TBIs [55,56]. In vitro studies have shown that progesterone in female rats has improved the outcome after TBIs compared with male rats [57]. Some studies have also shown that progesterone infusion after TBIs can reduce cerebral edema and secondary brain damage in both sexes of rats [58]. Progesterone can suppress the synthesis of inflammatory cytokines, including TNF-alpha, interleukins 1 and 6, microglial activation, and neuronal damage [59,60,61]. There is also evidence that progesterone reduces lipid peroxidation and free radical production and activates repair mechanisms to combat reactive oxygen species.

## 5. Limitations

Our main limitation is that sex hormone levels were only measured once after TBIs instead of conducting dynamic measurements over time. Furthermore, the study did not include MRI or EEG analyses to observe periodic changes, which could have provided valuable prognostic information about the patients. This research was explicitly designed to examine the role of sex hormones as predictive factors for the return of consciousness after TBIs and to explore the differences between the two sexes.

## 6. Conclusions

In conclusion, the sex hormone profiles in males and females exhibit notable differences during the acute phase of TBIs. Serum testosterone levels in males and progesterone levels in females serve as valuable prognostic indicators for evaluating the return of consciousness following TBIs. Consequently, patients need to undergo post-TBIs neuroendocrine assessments after achieving hemodynamic stability. In females, treatment with progesterone and estrogen effectively facilitates the return of consciousness. However, further studies are necessary to elucidate the mechanisms by which testosterone and progesterone influence recovery in both sexes. Future research should incorporate a larger sample size and MRI and EEG findings to monitor patient conditions, and dynamic measurements of sex hormones should be considered during the follow-up period.

## Figures and Tables

**Table 1:** Patients baseline characteristics.

Variable	Categories	Male (n = 60)	Female (n = 60)	*p*-Value
Age (years)	Mean ± SD	33.40 ± 6.52	34.25 ± 5.93	0.288
Mechanism of injury, (N, %)	Automobile	44 (73.4%)	38 (63.3%)	0.212
	Motorcycle	9 (15%)	8 (13.3%)	0.198
	Fall/Jump	7 (11.7%)	14 (23.3%)	0.319
Radiological type of injury, (N, %)	Subdural hematoma	20 (33.3%)	18 (30%)	0.549
	Diffuse axonal injury	7 (11.7%)	6 (10%)	0.910
	Epidural hematoma	5 (8.3%)	3 (5%)	0.489
	Subarachnoid hematoma	9 (15%)	10 (16.7%)	0.320
	Contusion	13 (21.7%)	15 (25%)	0.224
	Interventricular hematoma	3 (5%)	4 (6.7%)	0.768
	Intracerebral hemorrhage	3 (5%)	4 (6.7%)	0.768
GCS at admission	Mean ± SD	4.70 ± 1.18	4.51 ± 1.17	0.198
ISS	Mean ± SD	35.96 ± 3.53	36.03 ± 3.64	0.460
Time from injury to measure the level of sex hormone (days)	Mean ± SD	2.46 ± 1.09	2.60 ± 1.13	0.257

GCS: Glasgow Coma Scale; ISS: Injury Severity Score; SD: standard deviation. The GCS score ranges from 3 to 15, with lower values indicating a lower level of consciousness. The ISS score ranges from 0 to 75, with higher values indicating greater injury severity.

**Table 2:** Single-factor analysis of serum sex hormone levels between sex groups by recovery of consciousness status.

Hormone	RC Group			NRC Group		
	Male	Female	*p*-Value	Male	Female	*p*-Value
Testosterone (ng/mL)	2.56 ± 0.47	0.17 ± 0.08	*p* < 0.001	0.81 ± 0.41	0.20 ± 0.08	*p* < 0.001
Estradiol (pg/mL)	20.16 ± 7.41	80.96 ± 12.84	*p* < 0.001	34.21 ± 7.47	39.00 ± 13.70	0.099
FSH (mIU/L)	4.61 ± 1.37	12.66 ± 5.00	*p* < 0.001	1.93 ± 0.90	23.53 ± 2.71	*p* < 0.001
LH (mIU/L)	3.27 ± 1.18	6.88 ± 1.19	*p* < 0.001	4.73 ± 0.96	9.98 ± 1.62	*p* < 0.001
Progesterone (ng/mL)	0.15 ± 0.05	1.80 ± 0.31	*p* < 0.001	0.33 ± 0.06	0.62 ± 0.31	*p* < 0.001
Prolactin (ng/mL)	22.09 ± 6.13	60.90 ± 8.14	*p* < 0.001	33.94 ± 4.12	49.53 ± 12.10	*p* < 0.001

FSH: follicular stimulating hormone; LH: luteinizing hormone. The results are given as mean ± standard deviation.

**Table 3:** Logistic regression analysis of sex hormones predicting patient recovery of consciousness.

Variable	Multivariate Analysis		
	Odds Ratio	95% Confidence Interval	*p*-Value
**Male Patients**			
Testosterone (ng/mL)	4.798	2.121–8.318	<0.001
FSH (mIU/L)	0.309	0.109–2.476	0.120
LH (mIU/L)	0.412	0.009–1.194	0.212
Progesterone (ng/mL)	0.879	0.101–2.412	0.109
Prolactin (ng/mL)	1.033	0.204–1.203	0.178
**Female Patients**			
Testosterone (ng/mL)	1.217	0.708–3.309	0.289
FSH (mIU/L)	1.133	0.103–2.467	0.187
LH (mIU/L)	0.303	0.001–0.903	0.294
Progesterone (ng/mL)	7.930	0.303–10.190	<0.001
Prolactin (ng/mL)	0.178	0.004–0.890	0.133

FSH: follicular stimulating hormone; LH: luteinizing hormone.

## Data Availability

Data can be presented on request.
